# HOXA9 and CD163 potentiate pancreatic ductal adenocarcinoma progression

**DOI:** 10.1186/s13000-024-01563-5

**Published:** 2024-10-26

**Authors:** Aiat Shaban Hemida, Mohamed Mohamady Ahmed, Mona Saeed Tantawy

**Affiliations:** 1 Pathology Department, Faculty of Medicine, Menoufia University, Yassin Abd Elghafar Street, Shibin El Kom, Menoufia, 32511 Egypt; 2 Pathology Technician Fellow, National Liver Institute- Menoufia University, Shibin El Kom, Egypt; 3 Pathology Department, National Liver Institute- Menoufia University, Shibin El Kom, Egypt

**Keywords:** Pancreatic ductal adenocarcinoma, HOXA9, CD163

## Abstract

**Background:**

The role of HOXA9 requires investigations in pancreatic ductal adenocarcinoma (PDAC) as HOXA9 inhibitors are being developed. HOXA9 might attract CD163 expressed tumor associated macrophages (TAM) and could affect PDAC prognosis. This work aims to study the expression and relevance of HOXA9 and CD163 in PDAC progression.

**Materials and methods:**

Selected 98 PDAC and 98 adjacent non tumor tissues as a control group were immunostained with HOXA9 and CD163 antibodies.

**Results:**

PDAC displayed highly significant higher HOXA9 staining intensity, percent and H score values than control group. HOXA9 staining of PDAC cases showed significant associations with poor prognostic indicators including larger tumor size, higher grade and advanced stage. PDAC showed highly significant differences regarding CD163 macrophage-specific staining intensity, percent and H score values than control group. CD163 showed significant higher expressions with larger tumor size, higher histological grade and advanced stage group. HOXA9 staining in PDAC showed highly significant direct correlations with CD163 positive macrophages. Follow up of PDAC cases revealed that high median H score of HOXA9 and CD163 were significantly associated with worse overall survival. CD163 was an independent prognostic marker of worse survival.

**Conclusions:**

In conclusion, HOXA9 could potentiate PDAC progression by stimulating CD163 expressed TAM attraction in tumors. HOXA9 and CD163 could participate in PDAC therapy. HOXA9 and CD163 could be predictors of worse prognosis and shorter survival in PDAC.

**Supplementary Information:**

The online version contains supplementary material available at 10.1186/s13000-024-01563-5.

## Introduction

The seventh cause of cancer deaths is pancreatic ductal adenocarcinoma (PDAC). It is expected to become the second cause in Western countries [1]. Pancreatic cancer is known to have a poor prognosis which could be attributed to the shortage of effective therapeutic options [2]. Chemo-resistance represents a major problem facing locally advanced and metastatic cases. Trends towards studying characteristics of pancreatic cancers and its interaction with the nearby microenviroment in order to discover new therapeutic models are required to improve prognosis [3].

HOXA9 Functions as a transcription factor and is linked to proliferation, invasion, and metastasis of solid tumors such as colon cancer [4], nasopharyngeal carcinoma [5] and breast carcinoma [6]. Opposing study found that HOXA9 blocked breast cancer progression through BRCA1 modulation [7]. Therefore, HOXA9 might have dual functions as an oncogene or tumor suppressor gene depending on tumor heterogeneity. To our knowledge, only one previous study showed that the HOTTIP/WDR5/HOXA9/Wnt axis was involved in stemness of PDAC [8]. The role of HOXA9 requires further investigations in pancreatic cancer as HOXA9 inhibitors are being developed.

The crosstalk between inflammation and cancer was suggested when previous studies indicated an important role of tumor-associated macrophages (TAM) in tumor growth and metastasis [9, 10]. Tumor-associated macrophages are immunosuppressive cells which were involved in tumorigenesis and cancer progression and they also displayed a positive relationship with poor prognosis [11]. CD163 showed increased expression in TAM with increased resistance to therapy [12]. This work aims to study the expression and relevance of HOXA9 and CD163 in PDAC progression.

## Materials and methods

This case- control study retrospectively selected 98 PDAC and 98 adjacent non tumor tissues as a control group. Paraffin blocks and clinical data were gathered from Pathology Department, National Liver Institute, Menoufia University. The institutional ethical committee approved this work and IRB was obtained.

Histopathologic data of medical reports and examined hematoxylin and eosin stained slides were recorded. Histologic grading system of College of American Pathologists (CAP) was followed for tumor grading [13]. For statistical purposes, the cases were further grouped into low grade (GI and GII) and high grade (GIII) tumors. Staging of PDAC was reported based on the eighth edition of the American Joint Committee on Cancer (AJCC) staging system [14]. Lumping into early (T1 and T2) and advanced (T3 and T4) stage was done.

Tissue Microarray (TMA) blocks were constructed by a tissue arrayer’s needle set provided by the tissue microarrayer instrument manufacturing company (Breecher instrument Manual Microarray, Wisconsin, USA).

### Immunostaining

Immunostaining was done using an IgG anti- HOXA9 rabbit’s polyclonal antibody (0.1 mL concentrated and diluted 1:150) (Chongqing Biospes Co., Ltd, China. catalog# YPA2228) and Rabbit polyclonal antibody against CD163 (Chongqing Biospes Co., Ltd, China. cat. # YPA1450), 100 ul concentrated with dilution of 1:100 as primary antibodies. Antigen retrieval solution Tris-EDTA high PH was used (Dako, Ref K8000, Glostrup, Denmark). Positive control slides of prostatic cancer and lymph node for HOXA9 and CD163 were applied respectively. Negative control slides were incubated without adding the primary antibodies.

### Interpretation of immunostained slides

HOXA9 cytoplasmic or nucleocytoplasmic staining in any number of cells was designated as positive [15]. The positivity of CD163 was designated when cytoplasmic and/or membranous staining of cells was seen [16]. CD163 was evaluated in TAM as it was negative in tumor cells.

Intensity of the stain of HOXA9 and CD163 was weak, medium, or strong. Percentage of the stain of HOXA9 and CD163 was registered. H score was calculated using this equation;

H score = 1 x % of weakly stained cells + 2 x % moderately stained cells + 3 x % of strongly stained cells [17].

The median of H score values categorized cases as low and high groups.

### Analysis of survival data

The overall survival was estimated from the date of diagnosis to the date of death or last follow-up. Kaplan Meier survival curves were constructed to differentiate survival between compared groups using Log rank test. Multivariate cox regression analysis was used to detect factors affecting survival and the most independent factor or factors affecting survival.

### Statistics of the study

Statistics of the study were managed using Statistical Package for Social Science (SPSS) version 22 (SPSS Inc., Chicago, USA). Data were described using percentage, mean (χ2), standard deviation (SD) and median. Data were analyzed using Fisher’s exacts, Chi-square (χ2‐test), student t, Mann Whitney U (U test), Kruskal–Wallis, McNemar and Marginal Homogeneity Tests. Statistically significant P value was ≤ 0.05.

## Results

The studied cases included 98 PDAC and 98 control group. Males were 70 (71.4%) cases while 28 (28.6%) cases were females. Hepatitis C virus (HCV) infection was positive in 22 (22.4%) cases. Necrosis was seen in 17 (17.3%) cases. Tumor desmoplasia was evident in 53 (54%) cases. Ten cases (10.2%) were high grade while 88 (89.8%) cases were low grade tumors. Lympho-vascular invasion was present in 34 (34.7%) cases and perineural invasion was present in 96 (98%) cases. The resection margin was involved in 31 (31.6%) cases. Negative nodal status in 23 (23.5%) cases was recorded. Tumor size was less than 5 cm in about 85.5% of cases. Advanced T stage was in 39 (39.8%) cases (Table 1).

**Table 1:** Description of the studied PDAC cases according to clinicopathological parameters (*n* = 98)

		No.	%
Gender	Male	70	71.4
	Female	28	28.6
Virology (HCV infection)	Negative	76	77.6
	Positive	22	22.4
CA19.9 (*n* = 50)	Low (< 175)	25	50.0
	High (≥ 175)	25	50.0
Direct bilirubin (*n* = 90)	< 6	45	50.0
	≥ 6	45	50.0
Tumor recurrence (*n* = 38)	Absent	20	52.6
	Present	18	47.4
Necrosis	Absent	81	82.7
	Present	17	17.3
Epithelial Desmoplasia predominance	Desmoplasia	53	54.1
	Epithelial	38	38.8
	Equal	7	7.1
TIMCs	< 10	39	44.3
	≥ 10	49	55.7
Tumor Size (*n* = 97)	≤ 5	83	85.6
	> 5	14	14.4
Histologic grade	I	11	11.2
	II	78	79.6
	III	9	9.2
	Low	88	89.8
	High	10	10.2
LVI	Absent	64	65.3
	Present	34	34.7
Perineural invasion	Absent	2	2.0
	Present	96	98.0
Resection margin	Free	67	68.4
	Involved	31	31.6
N stage	N0	23	23.5
	N1	44	44.9
	N2	31	31.6
LN status	Negative	23	23.5
	Positive	75	76.5
T stage	T1	3	3.1
	T2	56	57.1
	T3	39	39.8
Stage grouping	Early	59	60.2
	Advanced	39	39.8

- CA19-9: Carbohydrate antigen 19 − 9 -HCV: Hepatitis C virus

- TIMCs: Tumor-infiltrating mononuclear cells - LVI: Lymphovascular invasion

-LN: Lymph node - T: Tumor stage

### Expression of HOXA9 in PDAC and control groups

**Fig. 1:**
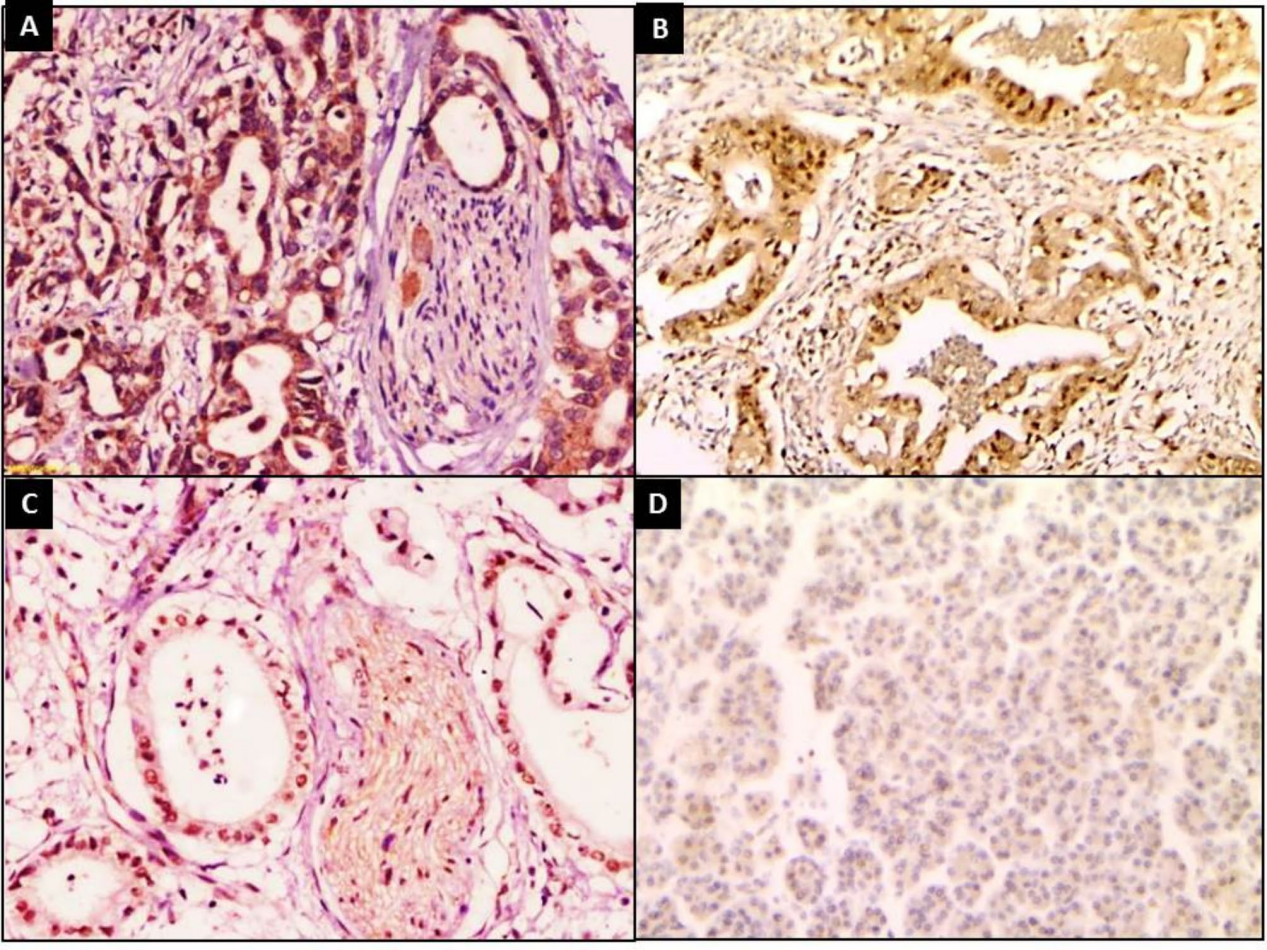
HOXA9 immunostaining showed (**A**) Strong expressions in PDAC with perineural invasion (**B**), (**C**) Moderate expressions in PDAC (IHCx200), (**D**) Mild expressions in control adjacent tissue (IHCx100)

All PDAC cases showed positive expression of HOXA9 (100%). Strong expressions were detected in 46 (46.9%) cases and moderate expressions were detected in 39 (39.8%) cases, while 13(13.3%) cases showed mild expressions. H score of HOXA9 staining ranged from 20 to 300 with 180.9 ± 79.1 as a mean ± SD and a median of 180 (Fig. 1).

HOXA9 staining was negative in 39 (39.8%) specimens of the control group. Mild expressions were detected in 45 (45.9%) and moderate expressions were detected in 14 (14.3%) specimens, while only one (1%) control group specimen showed strong HOXA9 staining. H score of HOXA9 staining ranged from 0 to 210 with 44.29 ± 46.79as a mean ± SD and a median of 30 (Fig. 1). PDAC and control groups displayed highly significant differences regarding HOXA9 staining intensity, percent and H score (*p* < 0.001) for all (Table 2).

**Table 2:** Comparison between PDAC and control groups regarding HOXA9 staining

	PDAC	Control	Test of Sig.	*p*
**H-score**				
Min. – Max.	20–300	0–210	Z = 8.459^*^	< 0.001^*^
Mean ± SD.	180.9 ± 79.1	44.29 ± 46.79		
Median (IQR)	180 (100–270)	30 (0–80)		
**Intensity**				
Negative	0 (0%)	38 (38.8%)	MH = 133.500^*^	< 0.001^*^
Mild	13 (13.3%)	45 (45.9%)		
Moderate	39 (39.8%)	14 (14.3%)		
Strong	46 (46.9%)	1 (1%)		
**Percentage**				
Min. – Max.	20–100	0–90	Z = 7.695^*^	< 0.001^*^
Mean ± SD.	74.80 ± 18.29	34.5 ± 32.2		
Median (IQR)	80 (60–90)	30 (0–60)		

SD: Standard deviation Z: Wilcoxon signed ranks test

MH: Marginal Homogeneity Test

p: p value for comparing between tumor and control

*: Statistically significant at *p* ≤ 0.05

### Relationship between HOXA9 staining and clinicopathological data of PDAC (*n* = 98)

**Fig. 2:**
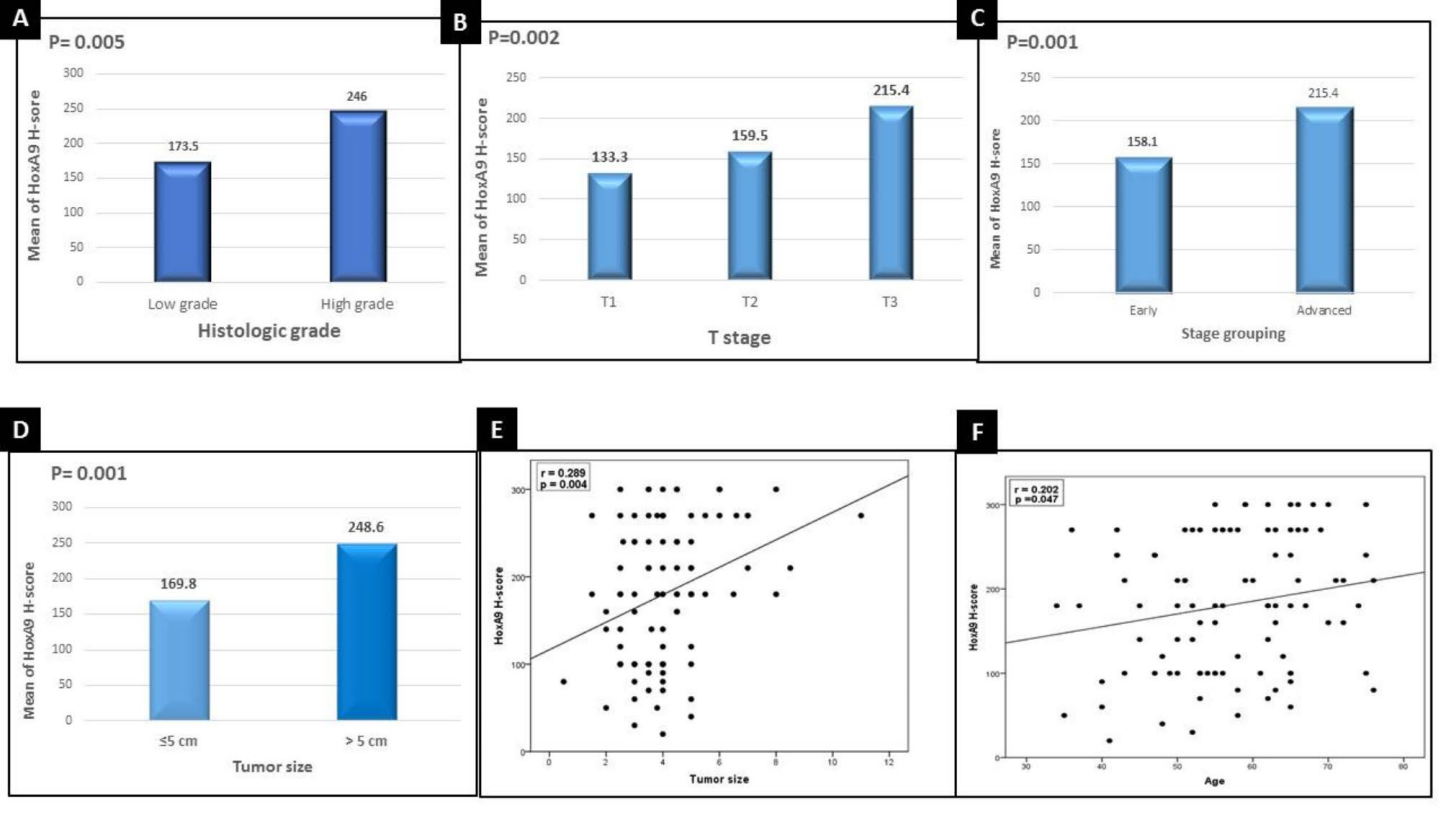
High H score values of HOXA9 staining showed significant associations with (**A**) higher histological grade (**B**) T2 tumor stage (**C**) advanced stage group (**D**) larger tumor size. HOXA9 staining showed significant direct correlations with (**E**) tumor size (**F**) patient older age

High H score values of HOXA9 staining showed significant associations with larger tumor size (*p* < 0.001), higher histological grade (*p* < 0.005), T2 tumor stage (*p* < 0.002) and advanced stage group (*p* < 0.001) (Fig. 2).

In addition, significant direct correlations between high H score values of HOXA9 staining and larger tumor size (*p* < 0.004) and older patient age (*p* < 0.047) were registered (Fig. 2).

### Expression of CD163 in PDAC and control groups

**Fig. 3:**
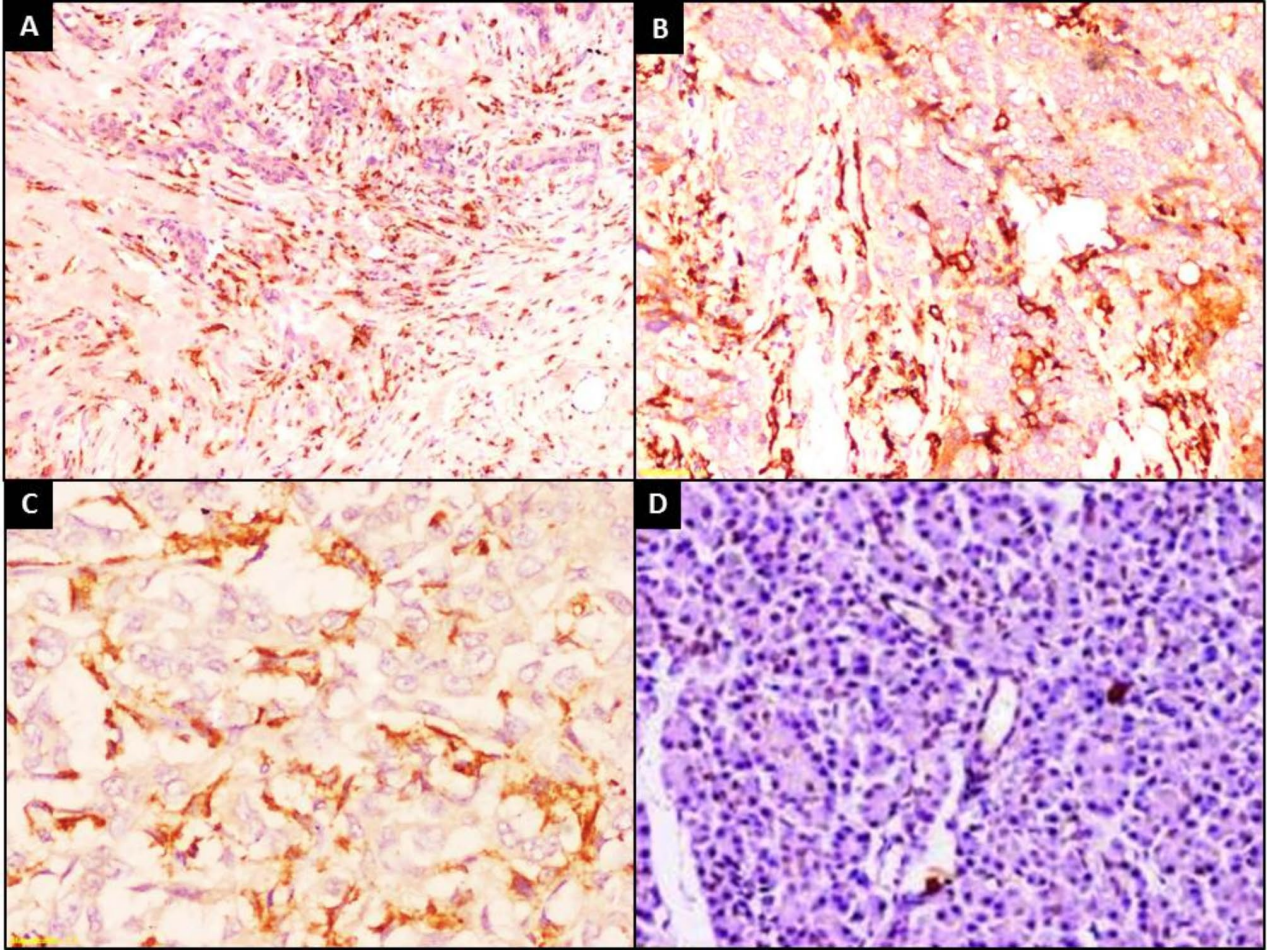
CD163 immunostaining showed that (**A**) PDAC Tumor cells were negative (IHCx40) (**B**) High expressions of CD163 in TAM in PDAC (IHCx200) (**C**) High expressions of CD163 in TAM in PDAC (IHCx400) (**D**) Negative expressions in control pancreatic tissue (IHCx200)

Tumor cells in all PDAC were negative for CD163 expression (100%). CD163 was positive in TAM in 91 (98.9%) of PDAC cases while in control group, it was positive in scattered macrophages in 37 (37.7%) specimens. Moderate expressions were detected in 45 (45.9%) of PDAC cases. While in control group, moderate expressions were detected in 2 (2%) specimens (Fig. 3).

H score of CD163 staining in TAM of PDAC ranged from 0 to 270 with 91.6 ± 56.1 as a mean ± SD of and a median of 100. In control group, H score of CD163 staining ranged from 0 to 160 with 15.10 ± 25.54 as a mean ± SD and a median of 0 (Fig. 3D).

PDAC and control groups displayed highly significant differences regarding CD163 staining intensity, percent and H score values (*p* < 0.001) for all (Table 3).

**Table 3:** Comparison between PDAC and control groups regarding CD163 staining

	PDAC	Control	Test of Sig.	*p*
**H- score**				
Min. – Max.	0–270	0–160	Z = 7.843^*^	< 0.001^*^
Mean ± SD.	91.6 ± 56.1	15.10 ± 25.54		
Median (IQR)	100 (40–120)	0 (0–30)		
**Intensity**				
Negative	7 (7.1%)	61 (62.2%)	MH = 89.500^*^	< 0.001^*^
Mild	37 (37.8%)	35 (35.7%)		
Moderate	45 (45.9%)	2 (2%)		
Strong	9 (9.2%)	0 (0%)		
**Percentage**				
Min. – Max.	0–100	0–90	Z = 7.645^*^	< 0.001^*^
Mean ± SD.	52.24 ± 224	13.88 ± 20.99		
Median (IQR)	60 (40–60)	0 (0–30)		

SD: Standard deviation Z: Wilcoxon signed ranks test

MH: Marginal Homogeneity Test

p: p value for comparing between tumor and control

*: Statistically significant at *p* ≤ 0.05

### Relationship between CD163 staining and clinicopathological data of PDAC (*n* = 98)

**Fig. 4:**
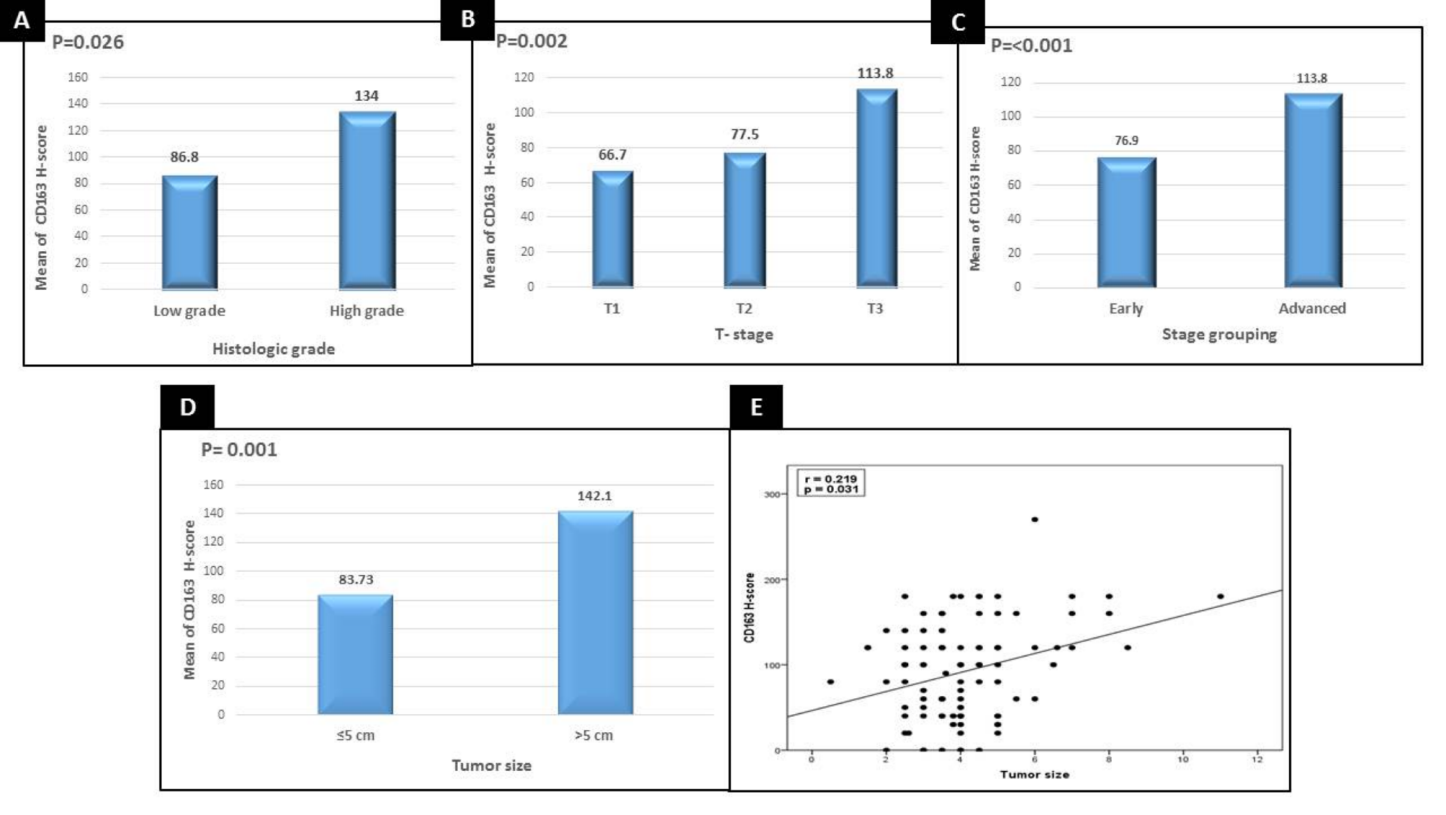
High H score values of CD163 staining showed significant associations with (**A**) higher histological grade (*p* < 0.026) (**B**) T3 tumor stage (*p* < 0.002) (**C**) advanced stage group (*p* < 0.001) (**D**) larger tumor size (*p* < 0.002) (**E**) Significant direct correlation between high mean H score value of CD163 staining with larger tumor size (*p* < 0.031)

High H score values of CD163 staining showed significant associations with larger tumor size (*p* < 0.001), higher histological grade (*p* < 0.026), T3 tumor stage (*p* < 0.002) and advanced stage group (*p* < 0.001) (Fig. 4).

In addition, a significant direct correlation between high mean H score value of CD163 staining with larger tumor size (*p* < 0.031) was registered (Fig. 4).

### Relationship between HOXA9 and CD163 expression in PDAC cases

A highly significant relationship between intensity of HOXA9 and CD163 staining in PDAC cases was detected. The nine cases (100%) that showed strong HOXA9 staining also showed strong CD163 staining (*p* < 0.001). Highly significant direct correlations between HOXA9 and CD163 as regards staining percent (rs = 0.424, *p* < 0.001) and H score values (rs = 0.571, *p* < 0.001) were also detected (Table 4).

**Table 4:** Relationships between HOXA9 and CD163 in PDAC (*n* = 98)

	*N*	HOXA9 (Intensity)				*p*
		Mild (*n* = 13)	Moderate (*n* = 39)	Strong (*n* = 46)		
**CD163 (Intensity)**						
Negative	7	1 (14.3%)	4 (57.1%)	2 (28.6%)	**FET** 30.394^*^	< 0.001^*^
Mild	37	12 (32.4%)	14 (37.8%)	11 (29.7%)		
Moderate	45	0 (0.0%)	21 (46.7%)	24 (53.3%)		
Strong	9	0 (0.0%)	0 (0.0%)	9 (100.0)		
HOXA9vs. CD163		**r**_**s**_				**p**
H- score		0.571^*^				< 0.001^*^
Percentage		0.424^*^				< 0.001^*^

r_s_: Spearman coefficient

*: Statistically significant at *p* ≤ 0.05

### Survival

Data about overall survival were collected for 53 out of the studied 98 cases (54.08%). Cases were followed-up from June 2008 to December 2023. The survival time ranged from 0.5 to 39 months with 11.99 ± 8.71 as a mean ± SD. Thirty seven PDAC cases died because of their disease (69.8%).

### Univariate and multivariate COX regression analysis for the parameters affecting overall survival of PDAC cases

**Fig. 5:**
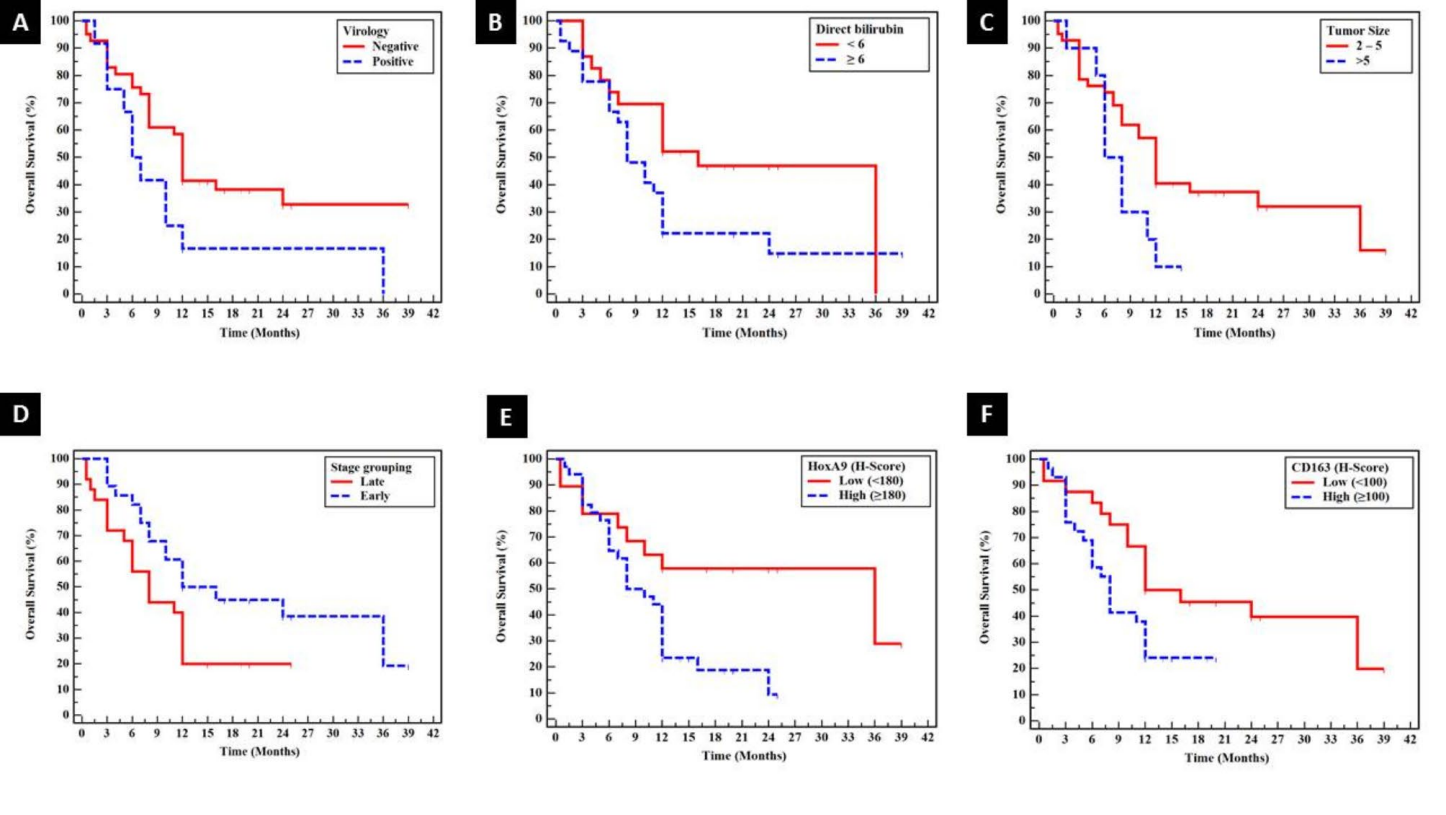
Shorter overall survival by univariate survival analysis of PDAC cases showed significant associations with (**A**) Positive virology (HCV infection) (*P* < 0.040) (**B**) high direct bilirubin (*P* < 0.046) (**C**) larger tumor size (*P* < 0.046) (**D**) advanced stage (*P* < 0.034) ((**E**) high median H score of HOXA9 (*P* < 0.015) and CD163 (*P* < 0.042)

Positive virology (HCV infection) (*P* < 0.040), high direct bilirubin (*P* < 0.046), larger tumor size (*P* < 0.046) and advanced stage (*P* < 0.034) showed significant associations with shorter overall survival by univariate survival analysis of PDAC cases. Shorter overall survival showed significant associations with high median H score of HOXA9 (*P* < 0.015) and CD163 (*P* < 0.042) (Table 5) (Fig. 5). Positive virology (HCV infection) (*p* < 0.016) was the most independent prognostic factor affecting overall survival for PDAC cases (Table 6).

**Table 5:** Univariate overall survival of PDAC cases (53 cases)

		*N*	% End of study	Mean (95% C.I)	SE	Log rank	*p* value
Gender	Male	39	14.3	17.07 (12.654–21.482)	2.252	0.248	0.619
	Female	14	35.7	10.43 (6.345–14.512)	2.084		
Virology (HCV infection)	Negative	41	32.8	18.81 (14.056–23.568)	2.426	4.201*	0.040*
	Positive	12	0.0	11.29 (4.499–18.084)	3.466		
CA19.9	Low (< 175)	16	43.8	20.59 (12.489–28.699)	4.135	0.878	0.349
	High (≥ 175)	11	0.0	12.23 (7.112–17.343)	2.610		
Direct bilirubin	< 6	23	0.0	21.174 (15.024 − 27.324)	3.138	3.975*	0.046*
	≥ 6	27	14.8	13.019 (8.258–17.779)	2.429		
Tumor recurrence	Absent	20	20.8	20.20 (13.115–27.285)	3.615	1.615	0.204
	Present	18	14.8	11.61 (8.577–14.645)	1.548		
Necrosis	Absent	42	15.3	17.12 (12.619–21.622)	2.297	0.051	0.821
	Present	11	27.3	12.55 (7.986–17.105)	2.326		
Epithelial Desmoplasia predominance	Desmoplasia	28	0.0	16.39 (11.226–21.544)	2.632	1.964	0.375
	Epithelial	18	38.9	14.28 (10.158–18.398)	2.102		
	Equal	7	14.3	10.00 (0.953–19.047)	4.616		
TIMCs	< 10	20	24.0	11.51 (7.925–15.095)	1.829	1.288	0.256
	≥ 10	31	15.5	17.87 (12.593–23.155)	2.694		
Tumor Size	2–5	42	16.0	17.88 (13.337–22.431)	2.320	3.969	0.046*
	> 5	10	10.0	7.85 (5.556–10.144)	1.171		
Histologic grade	I	8	25.0	15.75 (6.156–25.344)	4.895	0.076	0.963
	II	37	0.0	16.97 (12.274 − 21.664)	2.395		
	III	8	25.0	10.88 (7.104–14.646)	1.924		
	Low	44	15.6	17.31 (12.880 − 21.729)	2.257	0.227	0.634
	High	9	22.2	10.56 (7.152–13.959)	1.736		
LVI	Absent	34	18.5	17.997 (12.716 − 23.278)	2.694	0.278	0.598
	Present	19	13.2	12.684 (9.164–16.205)	1.796		
Perineural invasion	Absent	2	50.0	7.75 (0.000–17.798)	5.127	0.001	0.981
	Present	51	14.3	16.75 (12.779–20.717)	2.025		
Resection margin	Free	34	16.4	18.12 (13.154–23.091)	2.535	0.951	0.329
	Involved	19	26.3	10.29 (7.295–13.284)	1.528		
N stage	N0	14	28.6	11.54 (6.751–16.321)	2.441	0.375	0.829
	N1	24	22.2	16.75 (11.074–22.426)	2.896		
	N2	15	0.0	18.18 (10.242–26.113)	4.049		
LN status	Negative	14	28.6	11.54 (6.751–16.321)	2.441	0.372	0.542
	Positive	39	14.4	17.28 (12.706–21.852)	2.333		
T stage	T1	1	100.0	36.0 (36.0 – 36.0)	0.000	5.264	0.072
	T2	27	17.8	19.831(14.243–25.419)	2.851		
	T3	25	20.0	10.220 (6.977–13.463)	1.654		
Stage grouping	Early	28	19.3	20.557(15.029–26.085)	2.820	4.496	0.034*
	Advanced	25	20.0	10.220 (6.977–13.463)	1.654		
HOXA9 (H-Score)	Low (< 180)	19	28.9	24.026 (16.723 − 31.330)	3.726	5.948	0.015*
	High (≥ 180)	34	94.0	10.997 (8.446–13.548)	1.302		
CD163(H-Score)	Low (< 100)	24	19.9	20.881 (15.006 − 26.756)	2.998	4.130*	0.042*
	High (≥ 100)	29	24.1	9.741 (7.354–12.128)	1.218		

- CA19-9: Carbohydrate antigen 19 − 9 -HCV: Hepatitis C virus

- TIMCs: Tumor-infiltrating mononuclear cells - LVI: Lymphovascular invasion

-LN: Lymph node - T: Tumor stage

**Table 6:** Multivariate COX regression analysis for the parameters affecting overall survival

	*p* value	HR (LL – UL 95%C.I)
Virology (HCV infection) [Positive]	0.016^*^	2.741 (1.209–6.218)
Direct bilirubin [≥ 6]	0.077	2.048 (0.925–4.533)
Tumor Size [> 5]	0.420	1.512 (0.553–4.132)
Stage grouping [Late]	0.834	1.109 (0.422–2.915)
HoxA9 (H-Score) [High (≥ 180)]	0.424	1.492 (0.559–3.981)
CD163 (H-Score) [High (≥ 100)]	0.201	1.772 (0.737–4.264)

HR: Hazard ratio C.I: Confidence interval LL: Lower limit

UL: Upper Limit

#: All variables with *p* < 0.05 was included in the multivariate

*: Statistically significant at *p* ≤ 0.05

When using the studied variables as continuous values in the COX regression analysis, positive virology (HCV infection) (*p* < 0.0003), high direct bilirubin (*P* < 0.008) and high H-Score values of CD163 (*p* < 0.037) were the most independent prognostic factors affecting overall survival in PDAC cases (Table 7).

**Table 7:** Multivariate COX regression analysis for the parameters affecting overall survival (continuous variables)

	Multivariate	
	*p*	HR (LL – UL 95%C.I)
Virology [Positive]	0.003^*^	3.342 (1.487–7.510)
Direct bilirubin	0.008^*^	1.073 (1.019–1.131)
Tumor Size	0.521	0.918 (0.707–1.192)
Stage grouping [Late]	0.538	1.356 (0.514–3.578)
HoxA9 (H-Score)	0.366	1.002 (0.997–1.008)
CD163 (H-Score)	0.037^*^	1.009 (1.001–1.017)

HR: Hazard ratio C.I: Confidence interval LL: Lower limit UL: Upper Limit

*: Statistically significant at *p* ≤ 0.05

### Univariate and multivariate COX regression analysis for the parameters affecting overall survival in HCV negative PDAC cases

In HCV negative PDAC cases, shorter overall survival showed significant associations with high median H score of HOXA9 (*P* < 0.022) and CD163 (*P* < 0.015). None of the studied parameters were independent prognostic factor affecting overall survival for HCV negative PDAC cases (Table 8).

**Table 8:** Univariate and multivariate COX regression analysis for the parameters affecting overall survival in negative Virology (HCV) cases

	Univariate		^#^Multivariate	
	*p*	HR (LL – UL 95%C.I)	*p*	HR (LL – UL 95%C.I)
Direct bilirubin	0.079	1.048 (0.995–1.105)		
Tumor Size	0.280	1.135 (0.902–1.427)		
Stage grouping [Late]	0.232	1.608 (0.738–3.507)		
HoxA9 (H-Score)	0.022^*^	1.006 (1.001–1.012)	0.187	1.005 (0.998–1.012)
CD163 (H-Score)	0.015^*^	1.007 (1.001–1.013)	0.191	1.004 (0.998–1.011)

HR: Hazard ratio C.I: Confidence interval LL: Lower limit UL: Upper Limit

#: All variables with *p* < 0.05 was included in the multivariate

*: Statistically significant at *p* ≤ 0.05

## Discussion

HOXA9 was accused in tumor progression through multiple signaling pathways. Searching for the role of HOXA9 in PDAC is important as it may participate in PDAC therapy. However, direct inhibitors of HOXA9 haven’t been developed yet.

CD163 is a marker of TAM. Tumor-associated macrophages mediate tumor cells surveillance from immune system and chemo- resistance. CD163 positive TAM might be a promising prognostic indicator in pancreatic cancer especially when targeted immunotherapy is developed as it will tailor patient specific therapy [18, 19].

In this study, PDAC displayed highly significant higher HOXA9 staining intensity, percent and H score values than control group. Using immunohistochemical staining and qRT-PCR of HOXA9, Fu et al., 2017 confirmed increased expression of HOXA9 in 90 PDAC than adjacent non tumor tissue [8]. In addition, the expression of HOXA9 was also increased in colorectal cancers and nasopharyngeal carcinoma than control tissues [4, 5]. Moreover, it is known that hypoxic tumor environment is involved in carcinogenesis through activation of HOX enzymes [20]. Therefore, HOXA9 might enhance pancreatic cancer development.

In contrast, it was found that HOXA9 was reduced in cutaneous tumorigenesis. Decreased expressions of HOXA9 in non- melanoma skin cancer, including squamous cell carcinoma than control skin were registered [15, 21]. In addition, HOXA9 showed reduced expressions in breast cancer and its inhibition prevented cell growth [7]. Taken together, HOXA9 switches between these differential functions and mediates oncogenesis or tumor suppression. HOXA9 heterogenic role in carcinogenesis might depend on tumor type.

In this study, HOXA9 staining of PDAC cases showed significant associations with poor prognostic indicators including larger tumor size, higher grade and advanced stage. In addition, HOXA9 staining was significantly correlated with older patient age. Fu et al., 2017 found correlations between HOXA9 expressions and tumor grade, perineural invasion and nodal metastasis. However, no correlations with age or tumor size were detected [8]. In nasopharyngeal carcinoma, HOXA9 was associated with advanced clinical and T stage [5]. In ovarian cancer, significant associations between HOXA9 and presence of ascites and residual disease were detected. However, there were no significant associations with FIGO stage, tumor grade, lymphovascular invasion or patient age [22]. Therefore, HOXA9 might be an indicator of poor prognosis in PDAC and other cancer types.

In this work, PDAC displayed highly significant differences regarding CD163 macrophage-specific staining intensity, percent and H score values than control group. Previous study compared CD163 expression in pancreatic cancerous cells and para-cancerous cells and showed significant higher expressions in pancreatic cancer [23]. In addition, the serum level of soluble CD163 was higher in patients with PDAC compared to controls [24]. Therefore, CD163 macrophage-specific high expression might be implicated in initiation of pancreatic cancer.

In this study, CD163 showed significant higher expressions with larger tumor size, higher histological grade and advanced stage group. Shi et al., 2021 also found significant associations with high TNM stage in pancreatic cancer [23]. In pancreatic neuroendocrine tumors high CD163 positivity in macrophages showed a significant association with metastatic status [19]. In addition, CD163 expression on macrophages showed significant correlations with Duke’s stage, histologic grade and metastasis in colon cancer [25]. Furthermore, in sarcoma, soluble CD163 was associated metastatic disease and high-grade tumors [26]. Taken together, CD163 could predict poor prognosis of pancreatic cancer.

To our knowledge, the relations between HOXA9 and CD163 positive macrophages were not previously investigated in PDAC. In the present study, HOXA9 staining in PDAC showed highly significant direct correlations with CD163 positive macrophages. In ovarian cancer, high HOXA9 expression was associated with elevated M2 macrophages. This was confirmed when higher CD163 expressed macrophages were significantly associated with higher HOXA9 expressed tumors [27]. Similar results were reported in astrocytoma [28]. Taken together, HOXA9 stimulates TAM attraction in tumors which might be involved in tumor progression.

Follow up of PDAC cases revealed that shorter overall survival showed significant associations with positive virology (HCV infection), high direct bilirubin, larger tumor size and advanced stage. In multivariate COX regression analysis, positive HCV infection and high direct bilirubin were confirmed as independent prognostic markers of worse survival. The pathogenesis of pancreatic cancer associations with HCV and high bilirubin is still unclear. Egypt is one of the most affected countries of HCV infections which are hepatotropic viruses and have oncogenic properties. Anatomically, the pancreas is situated in the proximity of the liver with possibility of migration of HCV. Antigens of HCV were detected in the pancreas where they mediated chronic inflammation, metaplasia and malignant transformation. Another explanation is the common origin of hepatocytes and pancreatic cells in the multipotent endodermal cells [29]. HCV induced pancreatitis impaired exocrine pancreatic function and increased bilirubin [30]. Therefore, positive HCV infection and high direct bilirubin might be accused in PDAC progression and poor prognosis. These suggested modifiable risk factors might have a good impact on tumor prevention.

Cases with high median H score of HOXA9 and CD163 showed significant associations with worse overall survival. In multivariate COX regression analysis, CD163 was confirmed as an independent prognostic marker of worse survival; however HOXA9 was not an independent prognostic marker of worse survival. Similar to our results, Fei et al., 2020 confirmed these significant associations in pancreatic cancer [24]. In addition, Shi et al., 2021 found that patients with high CD163 expressions exhibited shorter overall survival and was an independent prognostic marker of worse survival [23]. In sarcoma, Univariate analysis revealed that soluble CD163 was a significant prognostic indicator of overall survival [26]. As regards association between HOXA9 and worse survival; Fu et al., 2017 detected negative association between HOXA9 and survival in pancreatic cancer [8]. In addition, HOXA9 mRNA expression in high grade ovarian serous cancer was associated with a low survival rate [22]. Moreover, similar findings in other tumors including glioblastoma [31] and acute myeloid leukemia [32] confirmed associations of HOXA9 with worse survival. Taken together, HOXA9 and CD163 are indicators of poor survival in PDAC.

In HCV negative PDAC cases, shorter overall survival showed significant associations with high median H score of HOXA9 and CD163 but they were not independent prognostic factor of worse survival in these patients. No previous studies investigated the effect of concomitant associations between HCV and these markers on PDAC prognosis. Taken together, the bad prognostic significance of HOXA9 and CD163 might be independent of associated HCV infection.

In conclusion, HOXA9 could potentiate PDAC progression by stimulating CD163 expressed tumor associated macrophages attraction in tumors. HOXA9 and CD163 could participate in PDAC therapy. HOXA9 and CD163 could be predictors of worse prognosis and shorter survival in PDAC.

### Limitations of the study

Funding was a limitation in this study. We could not add larger number of cases.

## Electronic supplementary material

Below is the link to the electronic supplementary material.

Supplementary Material 1

## Data Availability

No datasets were generated or analysed during the current study.

## Abbreviations

PDAC: Pancreatic Ductal Adenocarcinoma
TAM: Tumor Associated Macrophages
AJCC: American Joint Committee on Cancer
CAP: College of American Pathologists
TMA: Tissue microarray
