# Supplementary figures and images for: HOXA9 and CD163 potentiate pancreatic ductal adenocarcinoma progression
Source: Diagn Pathol. 2024 Oct 26;19:141. doi: 10.1186/s13000-024-01563-5 (PMC11514874; doi:10.1186/s13000-024-01563-5)

**Positive control photos**


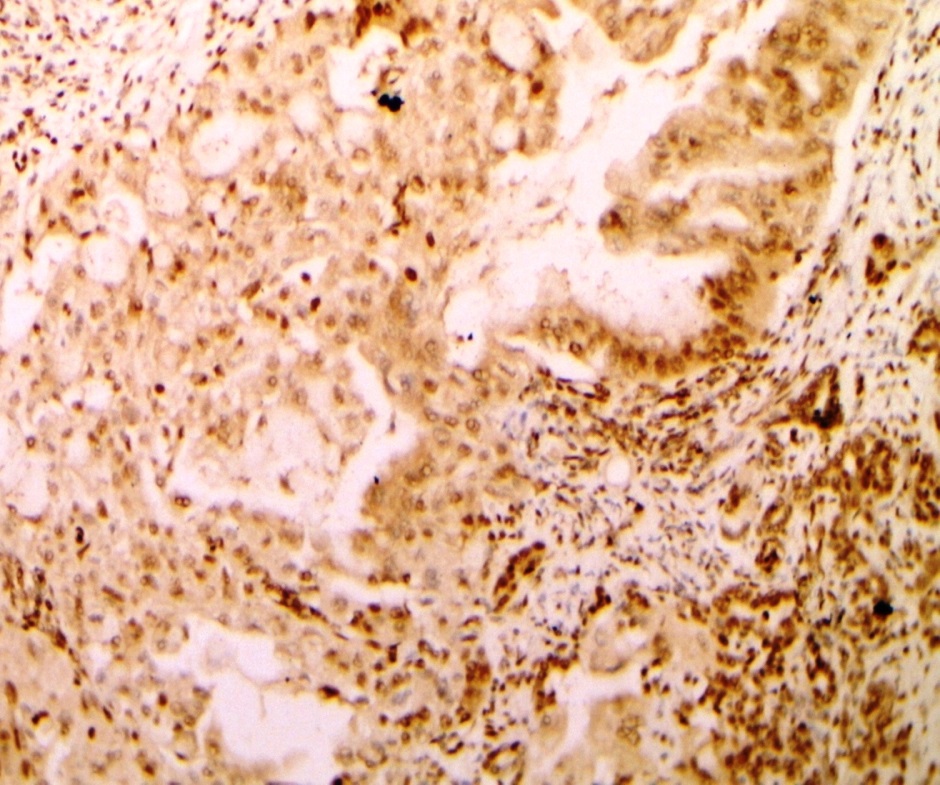


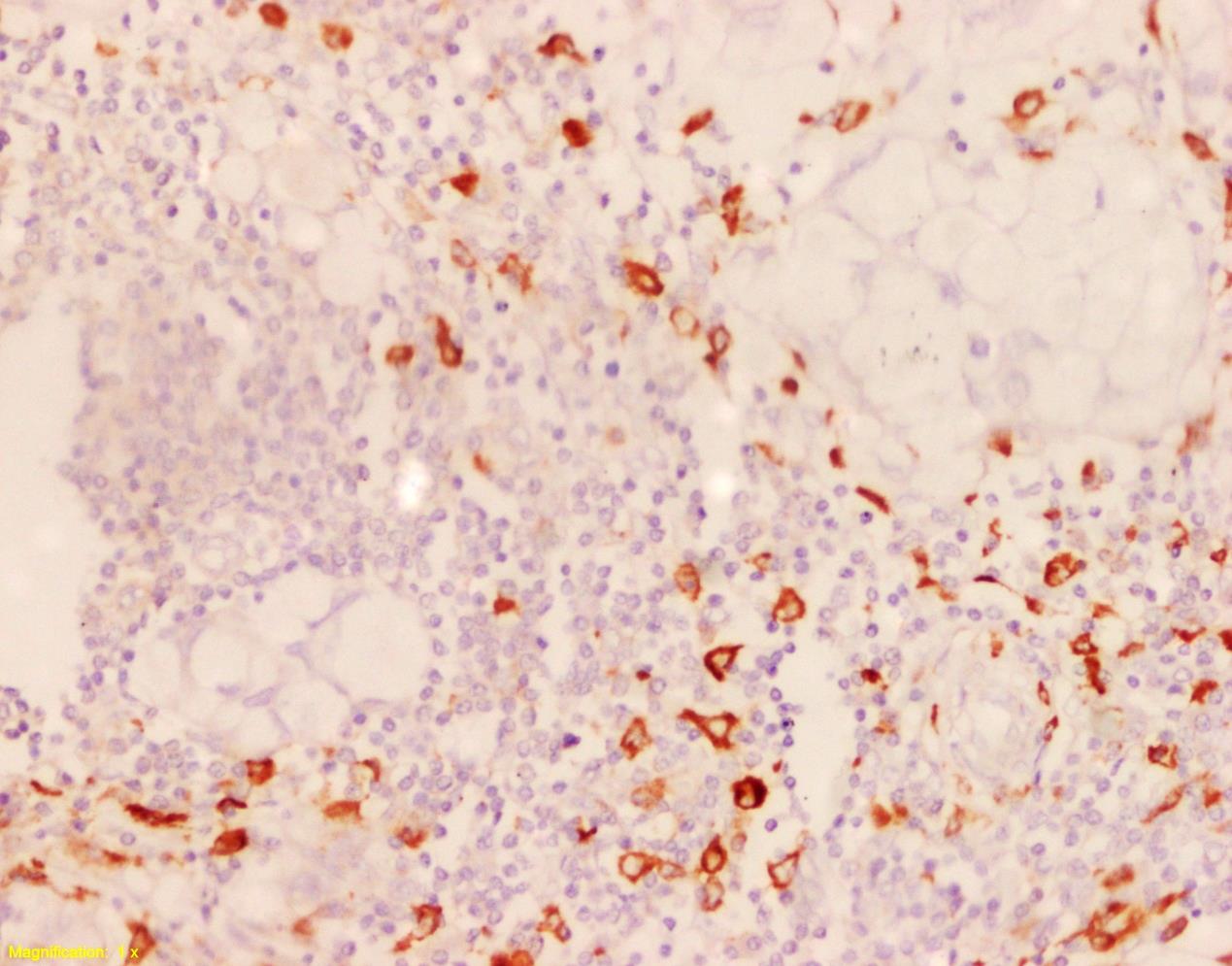

Supplement: Supplementary file 1 — Supplementary Material 1 [file 13000_2024_1563_MOESM1_ESM.docx]
